# Fractionation and phytochemical composition of an ethanolic extract of *Ziziphus nummularia* leaves: antioxidant and anticancerous properties in human triple negative breast cancer cells

**DOI:** 10.3389/fphar.2024.1331843

**Published:** 2024-02-09

**Authors:** Rola Abdallah, Abdullah A. Shaito, Adnan Badran, Serine Baydoun, Mansour Sobeh, Wafae Ouchari, Nihad Sahri, Ali H. Eid, Joelle Edward Mesmar, Elias Baydoun

**Affiliations:** ^1^ Department of Biology, American University of Beirut, Beirut, Lebanon; ^2^ Biomedical Research Center, Department of Biomedical Sciences at College of Health Sciences, and College of Medicine, Qatar University, Doha, Qatar; ^3^ Department of Nutrition, University of Petra, Amman, Jordan; ^4^ Breast Imaging Section, Imaging Institute, Cleveland Clinic Foundation, Cleveland, OH, United States; ^5^ Agrobiosciences Program, College for Agriculture and Environmental Science, Mohammed VI Polytechnic University, Ben Guerir, Morocco; ^6^ Department of Basic Medical Sciences, College of Medicine, QU Health, Qatar University, Doha, Qatar

**Keywords:** *Ziziphus nummularia*, oxidative stress, ROS, breast cancer, herbal medicine, apoptosis, autophagy, HPLC-PDA-MS/MS

## Abstract

Natural products have long been utilized in traditional medicine as remedies to improve health and treat illnesses, and have had a key role in modern drug discovery. Recently, there has been a revived interest in the search for bioactives from natural sources as alternative or complementary modalities to synthetic medicines; especially for cancer treatment, which incidence and mortality rates are on the rise worldwide. *Ziziphus nummularia* has been widely used in traditional medicine for the treatment of various diseases. Its traditional uses and numerous ethnopharmacological properties may be attributed to its richness in bioactive metabolites. However, its phytochemical composition or chemopreventive effects against the aggressive triple-negative breast cancer (TNBC) are still poorly explored. Here, phytochemical composition of an ethanolic extract of *Z. nummularia* leaves (ZNE) and its chromatographically isolated fractions was identified both qualitatively by spectrophotometric assays and analytically by HPLC-PDA-MS/MS. The anti-proliferative effects of ZNE were tested in several cancer cell lines, but we focused on its anti-TNBC effects since they were not explored yet. The anti-cancerous potential of ZNE and its fractions was tested *in vitro* in MDA-MB-231, a TNBC cell line. Results showed that ZNE and its Fraction 6 (F6) reduced the viability of MDA-MB-231 cells. F6 decreased MDA-MB-231 viability more than crude ZNE or its other fractions. ZNE and F6 are rich in phytochemicals and HPLC-PDA-MS/MS analysis identified several metabolites that were previously reported to have anti-cancerous effects. Both ZNE and F6 showed potent antioxidant capacity in the DPPH assay, but promoted reactive oxygen species (ROS) production in MDA-MB-231 cells; an effect which was blunted by the antioxidant N-acetyl cysteine (NAC). NAC also blunted ZNE- and F6-induced reduction in TNBC cell viability. We also demonstrated that ZNE and F6 induced an arrest of the cell cycle, and triggered apoptosis- and autophagy-mediated cell death. ZNE and F6 inhibited metastasis-related cellular processes by modifying cell migration, invasion, and adhesion. Taken together, our findings reveal that *Z*. *nummularia* is rich in phytochemicals that can attenuate the malignant phenotype of TNBC and may offer innovative avenues for the discovery of new drug leads for treatment of TNBC and other cancers.

## 1 Introduction

Plants were used as traditional remedies to prevent, relieve, and treat ailments since ancient times. Plants and botanical drugs or their metabolites were used in the treatment of common diseases including malaria, pneumonia, tuberculosis, among many others (Susana et al., 2019). Related to this study, many plant-derived metabolites were developed into important cancer chemotherapeutics such as paclitaxel, camptothecin, podophyllotoxin, and vincristine (Škubník et al., 2021; Asma et al., 2022; Majrashi et al., 2023). Cancer is still the second leading cause of disability and death globally, with around 19 million new cancer cases and almost 10 million deaths worldwide in 2020 (Sung et al., 2021). In addition, cancer is accompanied by a huge socio-economic burden to the patients, their families, and health systems (Alzehr et al., 2022). With the introduction of immunotherapy and other targeted cancer therapies, cancer treatments have advanced in the last few decades. However, surgery, chemotherapy, and radiotherapy remained the only treatment options in many cases. Radio- and chemo-therapy are accompanied by many therapy-induced side effects (Akram et al., 2017). They often cause damage to healthy tissues and can lead to nephro-, hepato-, neuro-, cardio-, and ototoxicity (van den Boogaard et al., 2022). Moreover, cancers can develop resistance to conventional treatments and relapse following remission. Therefore, there is an ongoing quest for new and effective cancer therapies including plant-derived therapies (Gezici and Şekeroğlu, 2019).

*Ziziphus nummularia* (Burm.fil.) Wight & Arn commonly called *Sidr*, is a spiny shrub that belongs to the Rhamnaceae family and grows mainly in arid and semi-arid regions (El Maaiden et al., 2020; Mesmar et al., 2022a). Cultures worldwide, particularly in India, Pakistan, China, and the Middle East including Iran and the Gulf countries, have long acknowledged the ethnopharmacological properties of the plant including health benefits, nutritional worth, and therapeutic attributes associated with species from the *Ziziphus* genus (Golla, 2018; Muhammad et al., 2020; Mesmar et al., 2022a). *Z. nummularia* is rich in bioactive phytochemical metabolites and a comprehensive analysis of its phytochemical composition has thus far identified approximately 431 chemical constituents belonging to alkaloids (mostly cyclopeptide alkaloids), flavonoids, terpenoids, saponins, as well as other minor phytochemicals like cholinergic acids, aromatic or polyaromatic metabolites, steroids, cerebrosides, and nucleosides (Mesmar et al., 2022a). Owing to their richness in these bioactive metabolites, plants of the *Ziziphus* genus have been described in the treatment of various conditions such as fever, diarrhea, skin infections and other skin conditions, conjunctivitis, helminthiasis, and gastric conditions, and have been reported to have antioxidant, anti-inflammatory, antimicrobial, and antinociceptive activities, among others (Chopra and Nayar, 1956; Upadhyay et al., 2011; Abbasi et al., 2013; Dey Ray et al., 2015; Hussain et al., 2017; Muhammad et al., 2020; Mesmar et al., 2022a). In the context of cancer, *Z. nummularia* plant extracts were found to attenuate the cancerous phenotype of Capan-2 human pancreatic cancer cells (Mesmar et al., 2021) and HeLa human cervical cancer cells (Beg et al., 2016). Dey Ray and Dewanjee showed that an ethanolic extract from the root bark of *Z. nummularia Aubrev* as well as a phytochemical isolated from this extract (termed isolated compound IC) were cytotoxic to MCF-7 human breast, K-562 leukaemia, OVCAR-3 ovarian, HT-29 colon, and A-498 kidny cancer cells. In addition, both of the extract and IC reduced tumor volumes and counts *in vivo* and increased the life span of female Swiss albino mice bearing Ehrlich ascites carcinoma (Dey Ray and Dewanjee, 2015). Lapachol, another phytochemical isolated from *Z*. *nummularia*, exhibited a remarkable antitumor activity *in vivo* against sarcoma-180 (S-180) cells engrafted into Swiss albino mice. In addition, lapachol sensitized the engrafted S-180 tumors to radiation (Kumar et al., 2002). These studies highlight the anticancerous potential and the richness of *Z. nummularia* in phytochemicals. These studies, in addition to the scarcity of studies on the potential therapeutic properties of this plant against breast cancer, prompted us to study the phytochemical composition of *Z. nummularia* and evaluate its effects *in vitro* on triple-negative breast cancer (TNBC), an aggressive type of cancer.

Breast cancer remains the most prevalent type of cancer in women (around 30%), affecting around 2.1 million women worldwide each year. In fact, breast cancer became the leading cancer type in the newly diagnosed cancer cases in 2020, surpassing lung cancer. Devastatingly, breast cancer is also the leading cause of cancer-related death in women and is the fifth leading cause of cancer-related deaths in both sexes (7%) in 2020 (Athamneh et al., 2020; World Health Organization, 2020; Sung et al., 2021; World Health Organization, 2021). TNBC has the worst prognosis and presents the greatest therapeutic challenge among the different subtypes of breast cancer. TNBC is highly invasive, and has a high potential to develop resistance to therapy and a significant rate of tumor relapse following treatment; TNBC tumor relapse rate is as high as 40% (So et al., 2022). TNBC accounts for 15%–20% of all breast cancers and lacks expression of estrogen receptor, progesterone receptor, and human epidermal growth factor receptor 2. These receptors normally allow breast cancer patients to respond to targeted and hormonal therapies. Consequently, their lack in TNBC makes conventional therapies (mainly surgery in combination with chemo- or radio-therapy) the only possible treatment options. TNBC patients are often non-responsive to therapy and the cancer can usually relapse post-treatment, with an average survival rate of only 10 months (Won and Spruck, 2020; Almansour, 2022). This mandates that alternative treatment approaches be sought. Relatedly, therapeutic strategies using plant sources have been gaining interest (Howes, 2018), and this study complements such strategies.

In this study, phytochemical compositions of an ethanolic extract from *Z. nummularia* (ZNE) and its fractions, obtained by chromatography, were evaluated both qualitatively by spectrophotometric assays and analytically by HPLC-PDA-MS/MS. The anti-cancerous potential of ZNE was investigated in a battery of cancerous cell lines but we focus our experiments on MDA-MB-231 cells, an aggressive TNBC cell line. Cytotoxicity of ZNE and its fractions towards MDA-MB-231 cells was tested using MTT assay. Plant extracts or their phytochemicals can suppress or induce oxidative stress, depending on their concentration (Chio and Tuveson, 2017; Alsamri et al., 2019; Shaito et al., 2020a; Huang R. et al., 2021). Therefore, DPPH radical scavenging assay was used to measure the antioxidant capacity of ZNE and Fraction 6 (F6) of the extract and 2′-7′-Dichlorodihydrofluorescein diacetate (DCFDA) was used to measure the effect of ZNE and F6 on reactive oxygen species generation in MDA-MB-231 cells. We also assessed the effect of ZNE and F6 on MDA-MB-231 cell cycle progression, hall marks of cancer metastasis (cell migration, aggregation, and adhesion), and apoptosis and autophagy processes.

## 2 Materials and methods

### 2.1 Collection of *Z. nummularia* leaves and preparation of their ethanolic extract (ZNE)

Leaves of *Z. nummularia* (Burm.fil.) Wight & Arn. were collected from south of Qatar (24.853,400, 51.274,767) during the period from April to May 2022. The plant was identified by Mohammad Al-Zein a resident plant taxonomist at the American University of Beirut (AUB) herbarium. The plant was identified according to index Kewensis as Kingdom: *Plantae*; Phylum: *Tracheophyta;* Class: *Magnoliopsida (*Dicotyledons); Order: R*osales*; Family: Rhamnaceae; Genus: *Ziziphus Mill*; Species: *Ziziphus nummularia*; Binomial name*: Z. nummularia* (Burm.fil.) Wight & Arn.

Leaves were washed and dried in the dark at room temperature and finely ground into a powder using a blender. The powder was suspended in 80% ethanol and incubated while shaking at 150 rpm in the dark for 72 h. Afterwards, the suspension was filtered using a filter paper, dried by a rotary evaporator, and lyophilized using a freeze dryer. The obtained powder was dissolved in 80% ethanol at a concentration of 100 mg/mL and stored in the dark at 4 °C.

### 2.2 Fractionation of ZNE crude extract

The ZNE crude extract was separated by a polyamide column chromatography system. ZNE was loaded onto a polyamide column and sequentially eluted using a gradient of water: ethanol (1:0, 9:1, 4:1, 3:2, 1:1, 1:4, 0:1), yielding seven distinct fractions. The fractions were evaporated using a rotary evaporator and freeze-dried. The obtained powders were stored at −20°C until their use.

### 2.3 Qualitative phytochemical analysis: total polyphenol content (TPC) and total flavonoid content (TFC)

The total polyphenol content (TPC) of ZNE was assessed using the Folin–Ciocalteu method with some minor adjustments (Mesmar et al., 2022b). ZNE was prepared at a concentration of 1 mg/mL. An aliquot of 500 μL from the extract was mixed with 2.5 mL of 0.2 N Folin–Ciocalteu reagent and allowed to oxidize for 5 min. The reaction was then neutralized by adding 2 mL of a 75 g/L sodium carbonate solution and subsequently incubated in the dark at 37°C for 1 h. After incubation, the absorbance of standards of a known polyphenol, gallic acid, and the samples was measured at 760 nm. TPC of ZNE and its fractions was expressed as a percentage of total gallic acid equivalents per gram of dry leaves used to make the extract (mg GAE/g). The TPC analysis was conducted in triplicates, and the results are presented as mean values ±SEM.

The total flavonoid content (TFC) of ZNE was determined through a modified aluminum chloride colorimetric assay (Mesmar et al., 2022b). Briefly, ZNE was prepared at a concentration of 1 mg/mL. An aliquot (1 mL) of this extract was then mixed with 1 mL of a 2% methanolic aluminum chloride solution. After incubation for 30 min in the dark at room temperature, the absorbance was measured at 415 nm, with quercetin serving as a standard. The TFC was quantified as mg quercetin equivalents per gram of dry leaves used to make the extract (mg QE/g). This analysis was performed three times, and the results are presented as mean values ± SEM.

### 2.4 HPLC-PDA-MS/MS

Analysis of the phytochemical composition of ZNE and its fractions was carried out using HPLC-PDA-MS/MS. A Shimadzu LC MS 8050 (Shimadzu, Japan) LC system was utilized alongside a triple quadruple spectrometer with an ESI source. A C18 reversed-phase column (Zorbax Eclipse XDB-C18, rapid resolution, 4.6 × 150 mm, 3.5 µm, Agilent, Santa Clara, CA, USA) was used for the separation process. Gradients of water and acetonitrile (ACN) in 0.1% formic acid were employed starting from 5% to 90% ACN over a period of 60 min with a flow rate of 1 mL/min. The injection of samples was automatically performed using an autosampler (SIL-40C xs) controlled by LC solution software (Shimadzu, Japan). The MS was operated in the negative ion mode.

### 2.5 Cell culture

MDA-MB-231 human breast cancer cells and other cell lines were obtained from ATCC (American Type Culture Collection, Manassas, VA). Cells were maintained in DMEM high-glucose medium supplemented with 10% fetal bovine serum (FBS; Sigma-Aldrich, St. Louis, MO, United States) and 1% penicillin/streptomycin (Corning, Massachusetts, United States) in a in a humidified chamber (37°C and 5% CO_2_).

### 2.6 MTT cell viability assay

MDA-MB-231 cells were seeded at a density of 5.0× 10^3^ cells/well of a 96-well tissue culture plate and allowed to grow until they reached 40% confluence. The cells were treated with increasing concentrations (0, 50, 100, 200, 400, and 600 μg/mL) of ZNE or F6 for a total period of 72 h. Cell viability was assessed by the reduction of 3-(4,5- dimethylthiazol-2-yl)-2,5-diphenyltetrazolium bromide (MTT; Sigma-Aldrich, St. Louis, MO, United States). Cell viability was determined as the proportional viability of the treated cells in comparison with the vehicle-treated cells (equivalent concentration of ethanol), the viability of which was assumed to be 100%.

### 2.7 DPPH (α, α-diphenyl-β-picrylhydrazyl) antioxidant activity assay

The antioxidant activity of ZNE and F6 was evaluated using the free-radical-scavenging activity of α, α-diphenyl-β-picrylhydrazyl (DPPH). Various concentrations of ZNE or F6 (50, 100, 200, 400, 600, or 800 μg/mL) were mixed with a solution of DPPH (0.5 mM in methanol). The blank solution, used for comparison, consisted of 80% ethanol (0.5 mL), DPPH solution (0.5 mL), and methanol (3 mL). Subsequently, the mixed samples were placed in darkness for 30 min, and their optical density (OD) was measured at a wavelength of 517 nm using a spectrophotometer. The percentage of DPPH-scavenging activity for each ZNE concentration was computed using the formula: % radical-scavenging activity = [(OD blank—OD plant extract at each concentration)/(OD blank)] × 100. Ascorbic acid was utilized as a standard for comparison.

### 2.8 ROS detection in cells by 2′-7′-dichlorodihydrofluorescein diacetate (DCFDA) staining

MDA-MD-231 cells were seeded in 12-well plates and grown until they reached 50% confluence. The cells were then treated with different concentrations (50, 100, or 200 μg/mL) of ZNE or F6 for 24 h. Afterwards, the medium was removed, and the cells were washed twice with 1X phosphate-buffered saline (PBS), and 2′-7′-Dichlorodihydrofluorescein diacetate (DCFDA) stain (10 μM) was then added to the cells. The cells were visualized using a ZEISS Axio Observer after 45 min.

In experiments involving N-acetyl cysteine (NAC; Sigma-Aldrich, St. Louis, MO, United States), a concentration of 10 mM NAC was added to the cells for 30 min prior to treatment with 100 μg/mL ZNE or F6. Cell viability was then determined at 24, 48, and 72 h using the MTT cell viability assay as described above.

### 2.9 Flow cytometry analysis of cell cycle

MDA-MB-231 cells were grown in 100 mm tissue culture plates for 24 h and then incubated in the presence or absence of different concentrations (50, 100, or 200 μg/mL) of ZNE or F6. Afterwards, cells were collected, washed twice, and suspended in 500 µL of phosphate-buffered saline (PBS). The cells were then fixed with an equal volume of 100% ethanol and maintained at a temperature of −20°C for at least 12 h. The cells were then pelleted, washed twice with PBS, resuspended in PBS containing DAPI (1 μg/mL), and incubated at room temperature for 30 min. The cell samples were analyzed using the BD FACSCanto II Flow Cytometry System from Becton Dickinson, with data collection facilitated by the FACSDiva 6.1 software.

### 2.10 Wound healing (scratch) assay

MDA-MB-231 cells were cultured in 12-well plates until they formed a confluent cell monolayer. Subsequently, a scratch was created across the confluent cell monolayer using a 10 μL pipette tip. The cell culture medium was removed, and the cells were rinsed with PBS to eliminate any debris. Fresh medium containing either the specified concentration (50, 100, or 200 μg/mL) of ZNE or F6 was then added, and cells were incubated at 37°C. Photomicrographs of the scratch were captured at baseline (0 h) and 12 h post-scratch using an inverted microscope (objective ×4). The width of the scratch was measured using the ZEN software (Zeiss, Germany) and expressed as the average difference ±SEM between the measurements taken at time zero and the indicated timepoint (12 h).

### 2.11 Trans-well migration and invasion assays

Trans-well inserts (8 μm pore size; BD Biosciences, Bedford, MA, United States) were used to evaluate the migratory ability of MDA-MD-231 cells. Briefly, cells were seeded at a density of 1.0 × 10^5^ cells per well into the upper chamber of the insert. The cells were then treated with either the vehicle control containing less than 1% ethanol or varying concentrations (50, 100, or 200 μg/mL) of ZNE or F6. The bottom wells of the system contained DMEM supplemented with 10% fetal bovine serum, serving as a chemo-attractant. Cells were incubated at 37°C for 24 h. Afterwards, the cells were washed, and non-penetrating cells were removed from the upper surface of the filter using a sterile cotton swab. Cells that had successfully migrated to reach the lower surface of the insert were fixed using 4% formaldehyde, stained with DAPI, and quantified using a fluorescence microscope. The assay was repeated three times and data were presented as mean values ±SEM.

The invasiveness of MDA-MB-231 cells was assessed in a trans-well migration chamber coated with Matrigel (8 μm pore size; BD Biosciences, Bedford, MA, United States). The experiment was performed like the trans-well migration assay, except that the cells need to invade through the Matrigel matrix to reach the lower surface of the insert. Cells that reached the lower surface of the insert were fixed using 4% formaldehyde, stained with DAPI, and quantified using a fluorescence microscope. The assay was repeated three times and data were presented as mean values ±SEM.

### 2.12 Gelatin zymography

MDA-MB-231 cells (1.0 × 10^6^) were cultured in 100 mm tissue culture plate in serum-free DMEM medium with or without different concentrations (50, 100, or 200 μg/mL) of ZNE or F6. After a 24 h incubation period, the conditioned media from the cultures were collected and concentrated. A total of 30 µg of proteins were separated using a 10% non-reducing polyacrylamide gel that contained 0.1% gelatin. Following electrophoresis, the gels were washed for 1 h in 2.5% (v/v) Triton X-100 to remove SDS, and then incubated at 37°C overnight in a solution comprising of 50 mM Tris-HCl (pH 7.5), 150 mM NaCl, 0.5 mM ZnCl_2_, and 10 mM CaCl_2_ to allow for the enzymatic degradation of the gelatin substrate by proteases in the media. The resulting gel was stained with 0.5% Coomassie brilliant blue R-250. Clear areas that appeared on the gel indicated gelatin degradation by matrix metalloproteinases (MMPs). Densitometry analysis was carried out using ImageJ software and the density of each cleared band was normalized to an equally loaded nonspecific band on the gel.

### 2.13 Adhesion assay

MDA-MB-231 cells were grown in the presence or absence of various concentrations (50, 100, or 200 μg/mL) of ZNE or F6 for a period of 24 h. They were then seeded onto 12-well plates that had been pre-coated with collagen and incubated at 37°C for 30 min. Afterwards, the cells were washed with PBS to eliminate non-adherent cells and the number of adherent cells was assessed using the MTT reduction assay.

### 2.14 Aggregation assay

Cell aggregation was assessed by collecting MDA-MB-231 cells from confluent plates using sterile 2 mM EDTA in Ca^2+^-/Mg^2+^-free PBS. These were aliquoted into separate non-adherent cell culture plates, with or without the treatment with ZNE or F6 (50, 100, or 200 μg/mL). Cells were incubated at 37°C with gentle shaking at 90 rpm for a period of 3 h and then fixed using 1% formaldehyde. Photomicrographs were captured for observation and analysis.

### 2.15 Whole cell protein extracts and Western blotting analysis

For the preparation of whole-cell lysates, MDA-MB-231 cells were washed twice with PBS and then lysed in lysis buffer containing 2% SDS and 60 mM Tris (pH of 6.8). The lysate mixture was subsequently centrifuged at 1.5 × 10^4^ g for 10 min. The protein concentration in the resulting supernatant was determined using a Bradford protein quantification kit (Biorad, Hercules, CA, United States). Aliquots of 25–30 μg of the protein extracts were resolved by 10% sodium dodecyl sulfate-polyacrylamide gel electrophoresis (SDS-PAGE) and then transferred onto a polyvinylidene difluoride membrane (Immobilon PVDF; Biorad). The PVDF membrane was blocked for 1 h at room temperature with a solution of 5% non-fat dry milk in TBST (Tris-buffered saline with 0.05% Tween 20). For immunodetection, the PVDF membrane was incubated overnight at 4°C with specific primary antibodies. The primary antibody was removed, the membrane washed with TBST, and then incubated with the secondary antibody, horseradish peroxidase-conjugated anti-IgG, for an hour. Following washing of the secondary antibody with TBST, immunoreactive bands were visualized using an enhanced chemiluminescence (ECL) substrate kit (Thermo Scientific, Rockford, IL, United States), following the manufacturer’s instructions. All primary and secondary antibodies used were obtained from Cell Signaling (Cell Signaling Technology, Inc., Danvers, MA, United States).

### 2.16 Statistical analysis

Results were evaluated using Student’s t-test. When comparing more than two means, one-way ANOVA followed by Dunnett’s *post hoc* test or two-way ANOVA followed by Tukey–Kramer’s *post hoc* test were also used. A *p*-value of <0.05 was considered as statistically significant.

## 3 Results

### 3.1 *Z. nummularia* leaves ethanolic extract and its fractions inhibit the proliferation of MDA-MB-231 breast cancer cells

**FIGURE 1:**
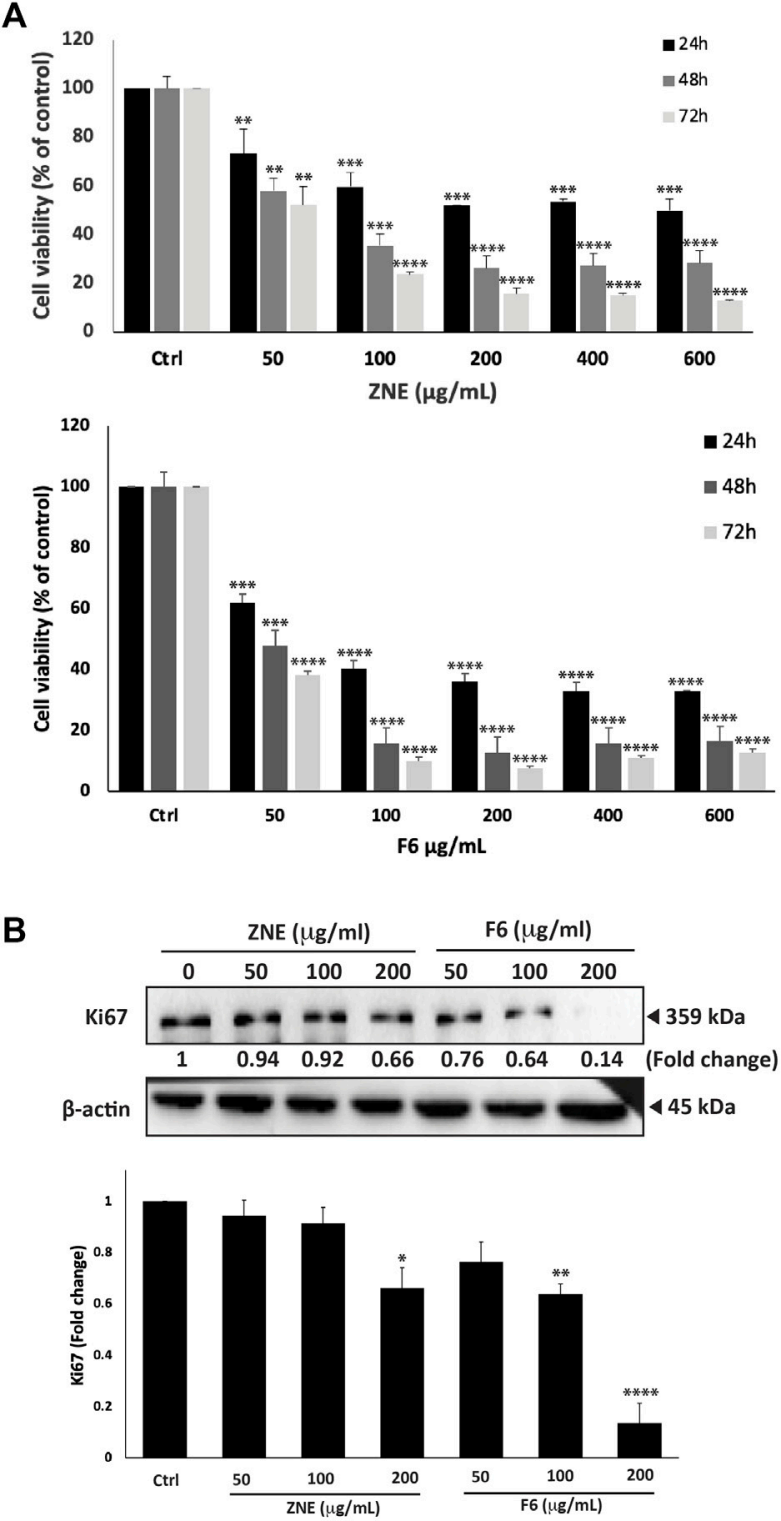
*Ziziphus nummularia* leaves ethanolic crude extracts inhibit proliferation of MDA-MB-231-cells. **(A)** MDA-MB-231 cells were treated the indicated concentrations of ZNE or F6 for 24, 48 and 72 h. Cell viability was determined using an MTT assay. Values are expressed as % viability compared to vehicle-treated control and are represented as the mean ± SEM of three independent experiments. **(B)** MDA-MB-231 cells were treated with indicated concentrations of ZNE or F6 for 24 h. Protein levels of Ki-67 were determined by Western blotting. Values are expressed as fold change of the vehicle-control and are represented as the mean ± SEM of three independent experiments. (*p < 0.05, **p < 0.01, ***p < 0.001, and ****p < 0.0001).

*Z. nummularia* ethanolic extracts showed an antiproliferative effect against several cell lines (data not shown), but we focused on MDA-MB-231 cells as a classical *in vitro* model of TNBC. To investigate the potential anti-proliferative property of ZNE on TNBC cells, the effect of various concentrations of the extract (0, 50, 100, 200, 400, and 600 μg/mL) were used to treat MDA-MB-231 cells for 24, 48, and 72 h. Results showed that ZNE treatment significantly attenuated cell viability in a time and concentration dependent manner. For example, at 48 h of treatment, a ZNE treatment at a concentration of 50, 100, 200, 400, or 600 μg/mL decreased viability of MDA-MB-231 cells to 57.9 ± 7.7, 35.3 ± 2.3 and 26.2 ± 6.0, 27.2 ± 4.4, 28.4% ± 3.0% that of control vehicle-treated cells, respectively (Figure 1A).

ZNE was separated into seven fractions using a polyamide column chromatography system. The effect of these seven fractions on MD-MB-231 cell viability was tested using the MTT assay. Fraction 6 (F6) reduced MDA-MB-231 cell viability to a significantly much higher extent than the rest of the fractions; therefore, F6 was used in the remaining experiments. The inhibitory effect of F6 was concentration- and time-dependent (Figure 1A). In fact, F6 showed a much stronger effect inhibitory effect than ZNE. For instance, at 48 h of treatment, cell viability using 50, 100, 200, 400, and 600 μg/mL of the F6 extract decreased to 47.7 ± 7.7, 15.8 ± 1.6, 12.9 ± 1.8, 15.8 ± 1.8, and 16.4% ± 2.3% that of control vehicle-treated cells, respectively (Figure 1A). The half-maximal inhibitory concentration (IC_50_) of ZNE on MDA-MB-231 cells was 662.4, 60.8, and 52.0 μg/mL at 24, 48, and 72 h, respectively while that of F6 was 68.5, 49.1, and 46.3 μg/mL at 24, 48, 72 h, respectively.

Western blotting of the cell proliferation marker Ki67, confirmed the results of MTT. Figure 1B shows that treatment of MDA-MB-231 cells with ZNE or F6 significantly reduced Ki67 protein levels in a concentration dependent manner. For example, 200 μg/mL of ZNE or F6 respectively caused 0.66-fold and 0.13-fold reduction in Ki-67 protein levels, compared to control vehicle-treated cells. ZNE and F6 extracts attenuate the cell proliferation process of MDA-MB-231 cells, with an enhanced effect observed with F6.

### 3.2 Phytochemical screening of ZNE and F6

Assays of TFC and TPC of ZNE and F6 showed that they are enriched in polyphenolic compounds and flavonoids. ZNE TPC was 664.708 ± 24.29 mg GAE/g and TFC of 25.57 ± 7.65 mg QE/g. F6 TPC was 154.90 ± 2.31 mg GAE/g and its TFC was 11.02 ± 1.01 mg QE/g.

### 3.3 Analysis of *Z. nummularia* extracts by LC-MS/MS

**FIGURE 2:**
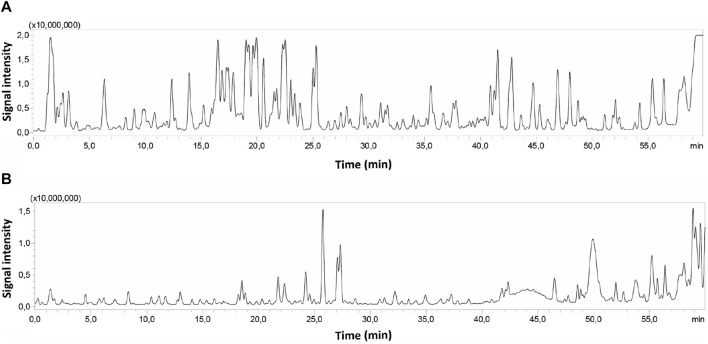
HPLC chromatograms of ZNE **(A)** and F6 **(B)** subjected to HPLC-PDA-MS/MS.

ZNE and F6 extracts were subjected to liquid chromatography analysis followed by MS/MS; HPLC-PDA-MS/MS. Their chromatographic profiles differed remarkably concerning the composition of their bioactive metabolites, with F6 showing less peaks than ZNE (Figure 2). For instance, 76 phytochemicals were present in ZNE, whereas F6 had 31 phytochemicals only (Table 1). For instance, F6 had high amounts of quercetin and kaempferol, which are also abundant in ZNE (Table 1).

**TABLE 1:** Annotated metabolites from ZNE and the F6 fraction using LC-MS/MS.

N	Rt (min)	MS/MS	[M-H] ^-^	Metabolite name	ZNE	F6
1	1.54	108, 191	191	Quinic acid	X	ND
2	1.72	115, 133	133	Malic acid	XXX	X
3	1.81	111	173	Shikimic acid	XXX	ND
4	2.34	111	191	Citric acid	XX	X
5	3.73	125	169	Gallic acid	X	ND
6	5.50	109,153	315	Protocatechuic acid glucoside	X	ND
7	6.11	125,179	305	Gallocatechin	XX	ND
8	6.56	109	153	Dihydroxybenzoic acid	XX	T
9	6.73	125, 305, 407	593	(epi)Catechin-(epi)gallocatechin	X	X
10	7.10	125, 305, 423	609	(epi)Gallocatechin-(epi) gallocatechin	X	ND
11	7.18	153, 182, 197	359	Syringic acid glucoside	X	ND
12	7.59	135, 191	353	Chlorogenic acid	X	ND
13	7.63	125, 305, 423	593	(epi)Catechin-(epi)gallocatechin	X	ND
14	7.87	125, 151, 169	315	Gallic acid pentoside	X	ND
15	8.33	108, 137	137	Hydroxybenzoic acid	ND	X
16	8.78	305	913	(epi)Gallocatechin-(epi) gallocatechin-(epi) gallocatechin	X	ND
17	8.82	125	577	(epi)Catechin-(epi)Catechin	X	T
18	9.64	119, 163, 191	337	Coumaroylquinic acid	X	ND
19	9.88	125,179	305	Gallocatechin	X	ND
20	10.46	135, 161	341	Caffeic acid glucoside	X	ND
21	10.66	119	325	Coumaric acid glucoside	X	X
22	10.713	125,179	465	Catechin glucuronide	X	ND
23	10.715		289	Catechin	X	ND
24	10.87	135, 161, 179	421	Caffeic acid glucoside sulfate	X	ND
25	10.99	135, 191	353	Neochlorogenic acid	X	X
26	11.62	121	177	Esculetin	X	ND
27	12.47	357, 387	597	Phloretin di-C-glucoside	XX	ND
28	13.82	119, 163, 191	337	Coumaroylquinic acid	XX	X
29	14.15	271, 317, 771	771	Myricetin dirahmnosyl glucoside	XX	ND
30	14.64	271, 317	757	Myricetin glucoside rhamnoside pentoside	X	ND
31	15.54	271, 317	611	Myricetin glucoside pentoside	X	ND
32	16.36	271, 301	755	Quercetin dirhamnosyl glucoside	X	ND
33	16.66	135, 179, 191	353	Cryptochlorogenic acid	XXX	ND
34	16.77	271, 317	625	Myricetin glucoside rhamnoside	XX	ND
35	17.31	119	163	Coumaric acid	XXX	ND
36	17.72	271, 301	771	Quercetin diglucoside rhamnoside	XX	ND
37	17.84	255, 285	739	Kaempferol glucoside dirhamnoside	XX	ND
38	18.58	125, 175, 285	303	Dihydro quercetin	ND	XX
39	18.58	271, 301	609	Quercetin glucoside rahmnoside	XXX	X
40	18.83	255, 285	725	Kaempferol glucoside rhamnoside pentoside	X	ND
41	19.07	271, 317	741	Myricetin dirhamnoside pentoside	XXX	ND
42	19.15	271, 317	595	Myricetin rhamnoside pentoside	XXX	ND
43	19.24	255, 285	593	Kaempferol glucoside rhamnoside	X	ND
44	19.77	271, 301	797	Quercetin dirhamnoside glucoside acetate	XXX	ND
45	20.67	255, 285	595	Kaempferol hydroxy coumaroyl glucoside	XX	ND
46	20.79	271, 317	609	Myricetin dirhamnoside	X	ND
47	21.41	255, 285	447	Kaempferol glucoside	XX	XX
48	21.65	271, 301	447	Quercetin rhamnoside	XXX	XX
49	21.87	271, 301	739	Quercetin trirhamnoside	XX	X
50	21.98	125, 169	477	Gallic acid glucoside rhamnoside	ND	XX
51	22.15	271, 301, 447	725	Quercetin dirhamnoside pentoside	XXX	X
52	22.35	271, 301, 447	579	Quercetin rhamnoside pentoside	XXX	X
53	23.05	271, 301, 477	901	Quercetin coumaroyl dirhamnosyl glucoside	XX	X
54	23.30	255, 285	709	Kaempferol rhamnosyl pentoside rhamnoside	X	X
55	23.58	271, 301, 447	593	Quercetin dirhamnoside	XXX	X
56	24.65	271, 301	463	Quercetin glucoside	X	X
57	24.94	255, 285	563	Kaempferol rhamnoside pentoside	XXX	ND
58	25.18	255, 285	431	Kaempferol rhamnoside	XXX	XX
59	25.60	119, 163	471	Coumaric glucoside rhamnoside	X	X
60	25.68		949	Unknown	ND	XXX
61	25.76	271, 315	461	Isorhamnetin rhamnoside	X	ND
62	25.96	285	475	Kaempferol dimethyl ether glucoside	X	ND
63	26.91	255, 301	755	Quercetin coumaroyl rhamnosyl glucoside	XX	X
64	27.15		1063	Unknown	X	XXX
65	27.19	135, 161	287	Caffeic acid derivative	X	XXX
66	27.94	337, 483	483	Coumaroylquinic acid rhamnoside	X	ND
67	29.25	151, 285	285	Kaempferol	XX	X
68	29.25	134, 247	533	Lotusanine A	X	ND
69	30.52	271, 301, 797	943	Quercetin coumaroyl dirhamnoside acetylglucoside	X	ND
70	30.97	285, 739	885	Kaempferol coumaroyl dirhamnosyl glucoside	X	ND
71	32.99	151, 271	271	Naringenin	X	ND
72	33.64	171, 211	327	Trihydroxy-octadecadienoic acid	X	ND
73	36.72	165, 183	227	Dihydroxy dodecanienoic acid	X	X
74	42.05	779, 895	941	Jujubasaponin IV/V	X	ND
75	44.39	749	911	Christinin A/C	XX	X
76	44.72	893, 953	997	Christinin A2	XX	ND
77	45.21	749	895	Jujubasaponin I	X	ND
78	45.50	749, 895	937	Jujubasaponin II/III	X	ND
79	52.06	423	485	Ceanothic acid	X	X
80	55.71	423, 467	485	Ceanothic acid isomer	X	X
81	59.03	423	499	Methyl ceanothate	XXX	ND

Rt: retention time, ND: not detected, T: traces, X: minor, XX: moderate, XXX: major, ZNE: *Z. nummularia* crude ethanolic extract, F6: Fraction 6.

### 3.4 ZNE and its fraction have high antioxidant capacity and increase the generation of ROS in MDA-MB-231 cells

**FIGURE 3:**
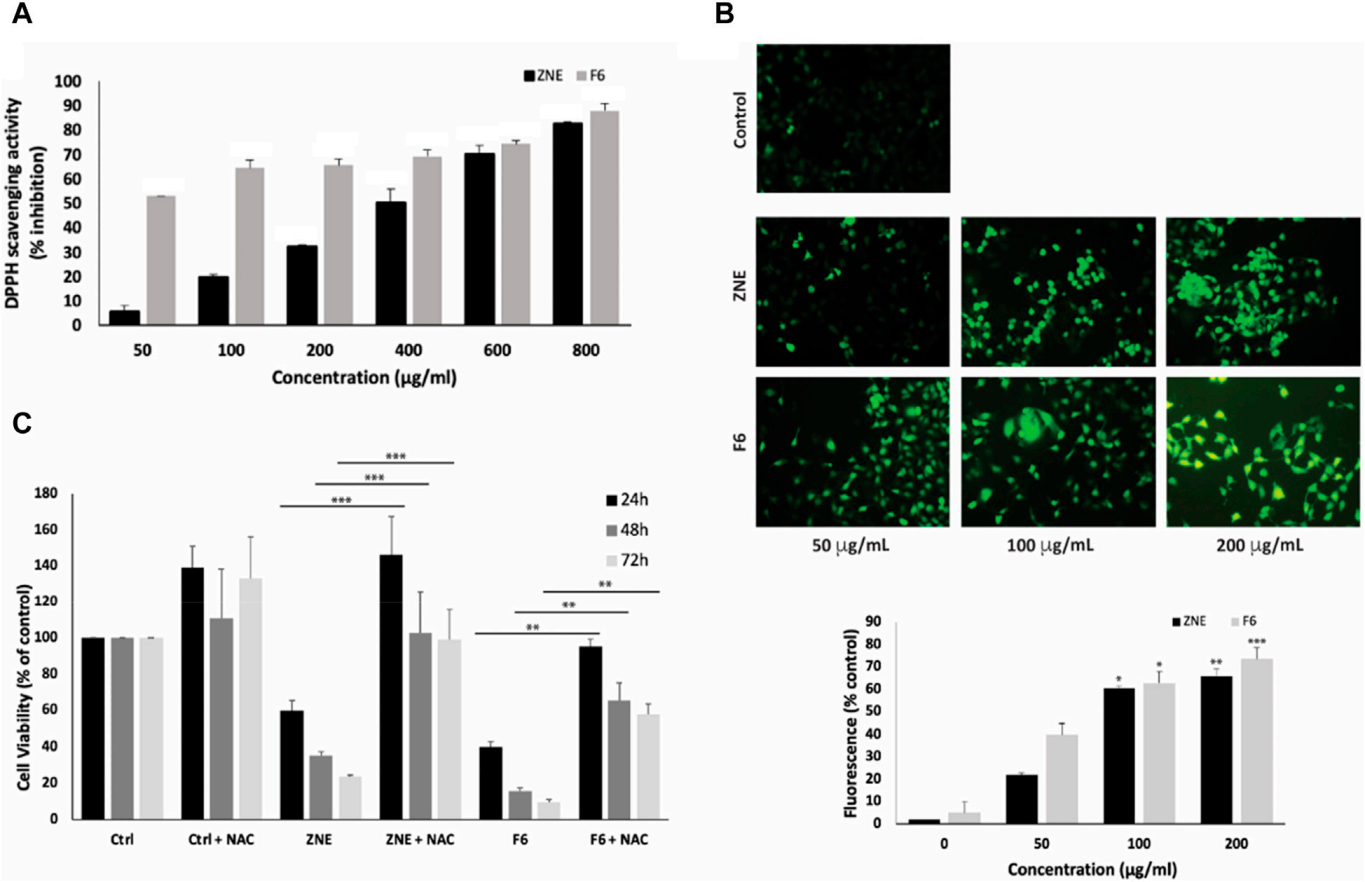
*Ziziphus nummularia* extracts have potent free-radical scavenging activity and increase ROS generation in MDA-MB-231 cells. **(A)** DPPH radical scavenging capacity assay was used to determine the antioxidant capacity of the indicated concentrations of ZNE or F6. Values are represented as the mean ± SEM of three independent experiments. **(B)** MDA-MB-231 cells were treated with the indicated concentrations of ZNE or F6 for 24 h and then stained with DCFDA to measure ROS production. Fluorescent images were then analyzed using ImageJ. Values are expressed as % of the control and are represented as the mean ± SEM of three independent experiments. **(C)** MDA-MB-231 cells were pre-treated with NAC (10 mM) for 30 min and then with ZNE (100 μg/mL) or F6 (100 μg/mL) for 24, 48 and 72 h. Cell viability was determined using an MTT assay. Values are expressed as % of the vehicle control and are represented as the mean ± SEM of three independent experiments. (**p < 0.01, ***p < 0.001, and ****p < 0.0001).

The antioxidant potential of ZNE and F6 was evaluated in the test tube using the DPPH-radical-scavenging assay. ZNE showed moderate free-radical-scavenging capacity, which was concentration-dependent. The IC_50_ of ZNE free-radical-scavenging activity was 373.13 μg/mL (Figure 3A). F6 exhibited a more pronounced free-radical-scavenging capacity with an IC_50_ of 32.88 μg/mL. This IC_50_ value was in the range of the free-radical-scavenging IC_50_ of the strong antioxidant ascorbic acid, indicating that F6 is rich in strong antioxidants.

Testing the effect of ZNE or F6 on ROS generation in MDA-MB-231 cells revealed that both ZNE and F6 increased the levels of ROS inside the cells in a concentration-dependent manner, as indicated by the increase in DCFDA fluorescence (Figure 3B).

To investigate whether the ZNE- or F6-induced generation of ROS is related to the anti-proliferative effects of the extracts, MDA-MB-231 cells were pretreated with N-acetyl cysteine (NAC), a ROS scavenger, prior to treatment with ZNE or F6. Results showed that NAC significantly attenuated ZNE and F6-mediated cell death (Figure 3C). For instance, the viability of cells treated with ZNE for 48 h was 35.3% ± 2.3% in the absence of NAC and was significantly rescued to 102.5% ± 23.1% when cells were pre-treated with NAC. Similarly, the viability of cells treated with F6 for 48 h was 15.8% ± 1.5% in the absence of NAC, and NAC pretreatment significantly elevated cell viability to 65.5% ± 9.6%. These results suggest that ZNE and F6 exert their anti-proliferative effect in TNBC cells, at least partly, through a ROS-dependent mechanism.

### 3.5 ZNE and F6 induces cell-cycle arrest of MDA-MB-231 cells at the G_1_ phase

**FIGURE 4:**
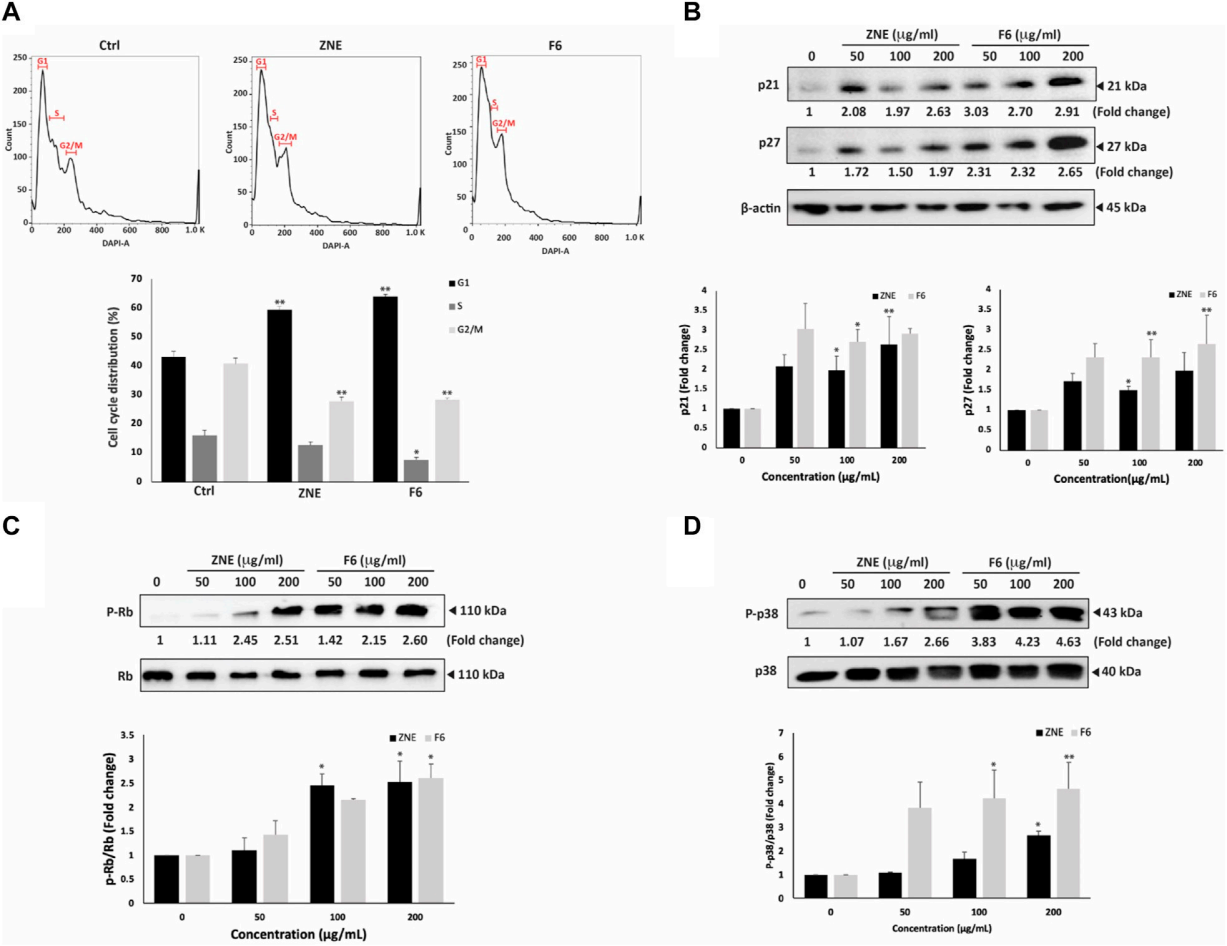
ZNE and F6 induce a cell cycle arrest at G1 phase in MDA-MB-231 cells. **(A)** MDA-MB-231 cells were treated with 100 μg/mL of ZNE or F6 for 24 h. Cells were then collected, fixed, stained with DAPI, and analyzed by flow cytometry as described in Materials and Methods. Values are expressed as the mean ± SEM of three independent experiments. **(B–D)** MDA-MB-231 cells were treated with indicated concentrations of ZNE or F6 for 24 h. Protein levels of phosphorylated P38 **(B)**, P21 and P27 **(C)** and RB and phosphorylated Rb **(D)** were then determined using Western blotting. Values are expressed as fold change of the control and are represented as the mean ± SEM of three independent experiments. (*p < 0.05, and **p < 0.01).

To further investigate the mechanisms of ZNE- and F6-induced reduction in viability of MDA-MB-231 cells, FACS analysis of the cell cycle distribution of MDA-MD-231 cells treated with ZNE or F6 was performed. ZNE and F6 induced an arrest of MDA-MB-231 cells at the G_1_ phase (Figure 4A). The percentage of cells in the G_1_ phase increased in ZNE- or F6-treated cells (59.4 ± 1.1 and 63.9 ± 0.8, respectively, *versus* 43.1 ± 1.8 in control cells). This was accompanied by a reduction in the percentage of cells in the S and G_2-_M phases, suggesting that ZNE and F6 trigger a cell cycle arrest at the G_1_ phase and inhibit cell entry into the S phase.

Since p38 MAPK (mitogen-activated protein kinase) pathway is commonly linked to inhibiting cell proliferation by controlling cell cycle progression and triggering apoptosis (Zhang and Liu, 2002), we analyzed the protein levels of the active phosphorylated form of p38 (P-p38) by Western blotting. Results show that the P-p38 levels were induced by ZNE and F6 in a concentration-dependent manner; 2.7 ± 0.2 and 4.6 ± 1.1- fold increase after treating the cells with ZNE or F6, respectively (Figure 4B). F6 showed a significant fold increase in P-p38 levels; 3.8 ± 1.1, 4.2 ± 1.2, and 4.6 ± 1.1-fold increase at 50, 100, and 200 μg/mL, respectively. While only the 200 μg/mL concentration of ZNE could significantly elevate P-p38 levels (2.7 ± 0.2-fold increase).

Furthermore, ZNE and F6 significantly increased the levels of the downstream effectors of p38, CDK inhibitors p21 and p27 (Figure 4C).

Retinoblastoma protein (Rb) is also a downstream effector of p38 signaling and has roles in tumor suppression, cell cycle control, differentiation, and apoptosis regulation (Du and Searle, 2009; Martínez-Limón et al., 2020). ZNE and F6 significantly increased the levels of P-Rb in a concentration-dependent manner (Figure 4D). Indeed, 200 μg/mL treatments of ZNE or F6 induced Rb phosphorylation by 2.5 ± 0.4 and 2.6 ± 0.3-fold, respectively, of the vehicle-control. F6 showed a stronger effect at lower concentrations with an observed 1.4 ± 0.3 and 2.1 ± 0.0-fold increases at the 50 and 100 μg/mL concentrations, respectively. These results further confirm the effect of ZNE and F6 on cell cycle dynamics.

### 3.6 ZNE and F6 extracts induce autophagy and apoptosis in MDA-MB-231 cells

**FIGURE 5:**
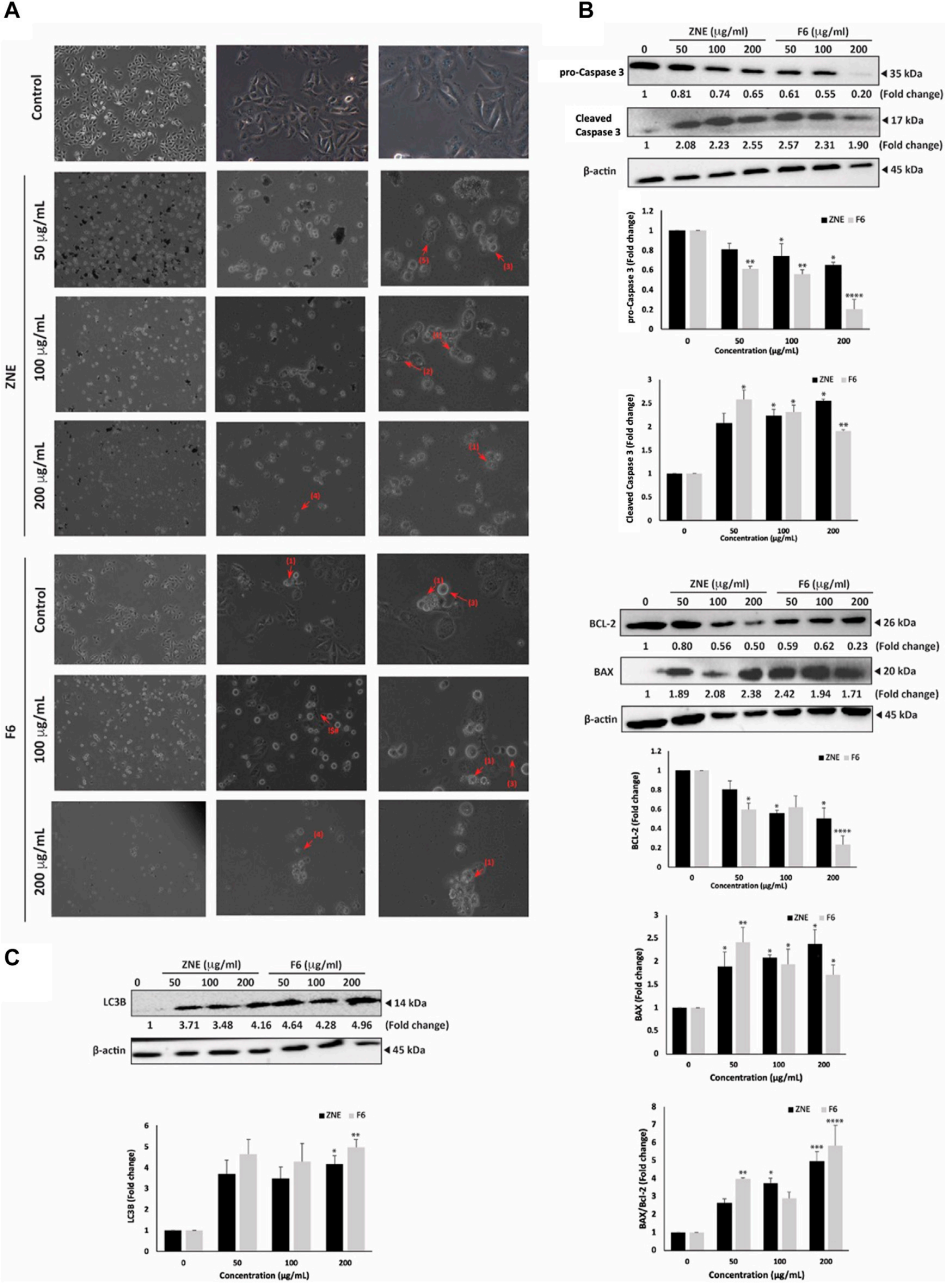
*Z. nummularia* extracts ZNE and F6 induce apoptosis and autophagy in MDA-MB-231 cells. **(A)** MDA-MB-231 cells were treated with indicated concentrations of ZNE or F6 for 24 h and microscopic images were acquired. Arrows indicate: (1) apoptotic bodies, (2) echeneid spikes, (3) membrane blebbing, (4) cell shrinkage, and (5) cytoplasmic vacuolation. **(B–D)** MDA-MB-231 cells were treated with indicated concentrations of ZNE or F6 for 24 h. Protein levels of pro-caspase 3 and cleaved caspase **(B)**, BCL-2 and BAX **(C)** and LC3B **(D)** were determined using Western blotting. Values are expressed as fold change of vehicle-control cells and are represented as the mean ± SEM of three independent experiments. (*p < 0.05, **p < 0.01, ***p < 0.001, and ****p < 0.0001).

Light microscopy of cells treated with ZNE or F6 revealed not only a concentration-dependent reduction in cell count per microscopic field, but also the presence of apoptotic bodies in the treated cells. Indeed, ZNE- or F6-treated cells showed cytoplasmic shrinkage, membrane blebbing, and apoptotic bodies (Figure 5A). Cytoplasmic vacuolation was also observed in treated cells, suggestive of autophagy in these cells. Notably, F6 treated cells were rich in these characteristics.

To confirm activation of apoptosis by ZNE and F6, protein levels of apoptosis effector enzyme pro-Caspase 3 and its cleavage products were examined by Western blotting. Pro-Caspase-3 protein levels were significantly attenuated in a concentration-dependent manner by ZNE or F6 treatment. For ZNE treatment, the decrease in pro-Caspase 3 protein levels was not significant at 50 μg/mL, but it was significant at concentrations of 100 and 200 μg/mL. While F6 significantly decreased the levels of pro-Caspase 3 at the 3 tested concentrations. At 200 μg/mL of ZNE or F6 there were 0.65 ± 0.02 and 0.20 ± 0.09-fold decrease, respectively (Figure 5B). Concomitantly, ZNE and F6 treatment significantly increased the levels of cleaved Caspase 3 fragments (Figure 5B). The increase in Caspase 3 cleavage products was significant at 100 and 200 μg/mL of ZNE, while it was significant for all the tested concentrations of F6. This suggests that *Z. nummularia* extracts induced the proteolytic cleave of pro-Caspase 3 into its active form caspase 3, thereby triggering the intrinsic apoptosis cascade.

Apoptosis is also regulated by the B-cell lymphoma (BCL-2-2) family of proteins. BCL-2 is involved in cell survival and inhibits apoptosis. While Bcl-2-associated X (BAX), another member of this family, accelerates apoptosis when overexpressed (Qian et al., 2022). Figure 5C, shows that cells treated with ZNE or F6 exhibited a decrease in BCL-2 levels and an increase in BAX levels in a concentration-dependent manner, indicative of the activation of intrinsic apoptosis upon ZNE or F6 treatment.

Finally, in order to determine whether autophagy was induced in ZNE and F6 treated cells, we examined the protein levels of LC3B, a marker of autophagosome formation (Runwal et al., 2019). Results indicated a substantial accumulation of LC3B in treated cells (Figure 5D). Taken together, ZNE, and particularly F6, decrease the viability of TNBC through several mechanisms including ROS generation, intrinsic apoptosis pathways, and the induction of autophagy.

### 3.7 ZNE and F6 reduce the migratory and invasive potential of MDA-MB-231 in a process involving MMP-9 downregulation

**FIGURE 6:**
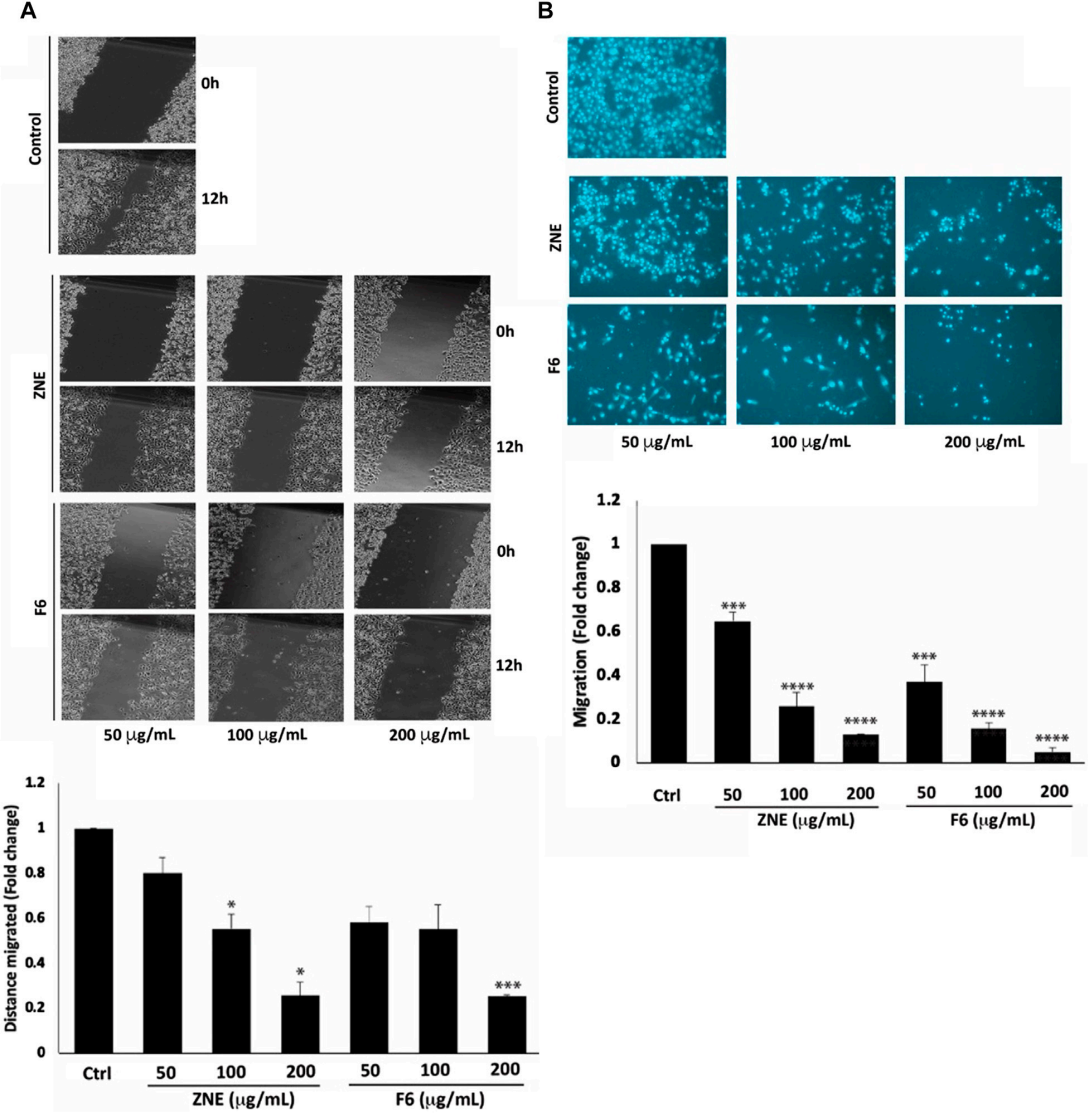
*Z nummularia* extracts ZNE and F6 reduce migration of MDA-MB-231 cells. MDA-MB-231 cells were treated with the indicated concentrations of ZNE or F6 and cell migration was assessed using a scratch/wound healing assay **(A)** and a Trans-well migration chamber assay **(B)**. Migratory cells were stained with DAPI and visualized using a fluorescence microscope. Values are expressed as fold change of the control and are represented as the mean ± SEM of three independent experiments. (*p < 0.05, ***p < 0.001, and ****p < 0.0001).

The early phases of cellular invasion and cancer metastasis involve modulation of cell migration. The results of a wound healing assay showed that ZNE and F6 significantly decreased the migration potential of MDA-MB-231 cells (Figure 6A). Cells treated with 100 and 200 μg/mL of ZNE showed a significant decrease in the distance migrated, while F6-induced decrease in migrated distance was significant only at the concentration of 200 μg/mL. The fold decrease in cell migration in cells treated with 200 μg/mL of ZNE or F6 was 0.26 ± 0.06 and 0.25 ± 0.01-fold that of vehicle-treated control cells, respectively. A trans-well migration assay confirmed these results. ZNE or F6 treatment caused a significant reduction in the number of cells crossing from the upper to the lower chamber at all the tested concentrations of 50, 100, 200 μg/mL (Figure 6B).

**FIGURE 7:**
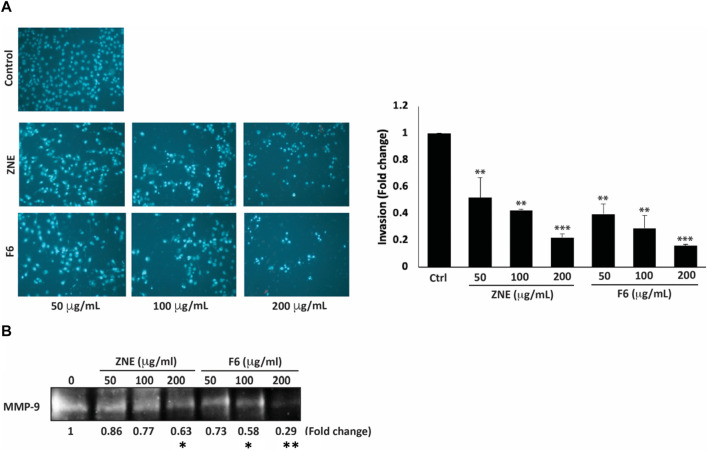
*Z. nummularia* extracts ZNE and F6 reduce the invasive potential of MDA-MB-231 cells. **(A)** MDA-MB-231 cells were treated with the indicated concentrations of ZNE or F6 and allowed to migrate through Boyden chamber trans-well inserts pre-coated with Matrigel. After 24 h, invading cells were stained by DAPI and visualized using a fluorescence microscope. Values are expressed as fold change of the control and are represented as the mean ± SEM of three independent experiments. **(B)** MDA-MB-231 cells were seeded in serum-free media and treated with the indicated concentrations of ZNE or F6. Conditioned media were concentrated and subjected to gelatin zymography to measure the activity of MMP-9 (**p < 0.01 and ***p < 0.001).

Increased cell migration along with an enhanced invasive ability is a hallmark of metastatic cancer cells, allowing them to migrate from primary tumor sites to invade secondary sites. ZNE and F6 significantly attenuated the invasive ability of MDA-MB-231 cells in a concentration-dependent manner; with F6 exhibiting a more pronounced effect. Indeed, a treatment of 200 μg/mL of ZNE or F6 significantly reduced the number of cells invading the Matrigel-coated membrane by 0.22 ± 0.02 and 0.16 ± 0.01-fold, respectively compared to vehicle-treated control cells (Figure 7A).

The degradation of the extracellular matrix (ECM) by matrix metalloproteinases (MMPs) is a widely recognized mechanism that promotes the migration and invasion of cancer cells (Lu et al., 2011). ZNE or F6 remarkably decreased MMP-9 activity levels at all the tested concentrations. At 200 μg/mL of ZNE or F6 the decrease was 0.46- and 0.45-fold, respectively, compared to vehicle-treated cells (Figure 7B). These data indicate that ZNE and F6 might reduce the invasive potential of MDA-MB-231 cells through inhibition of MMPs.

### 3.8 *Z. nummularia* extracts ZNE and F6 inhibit adhesion to collagen and downregulates integrin-β1 in MDA-MB-231 cells

**FIGURE 8:**
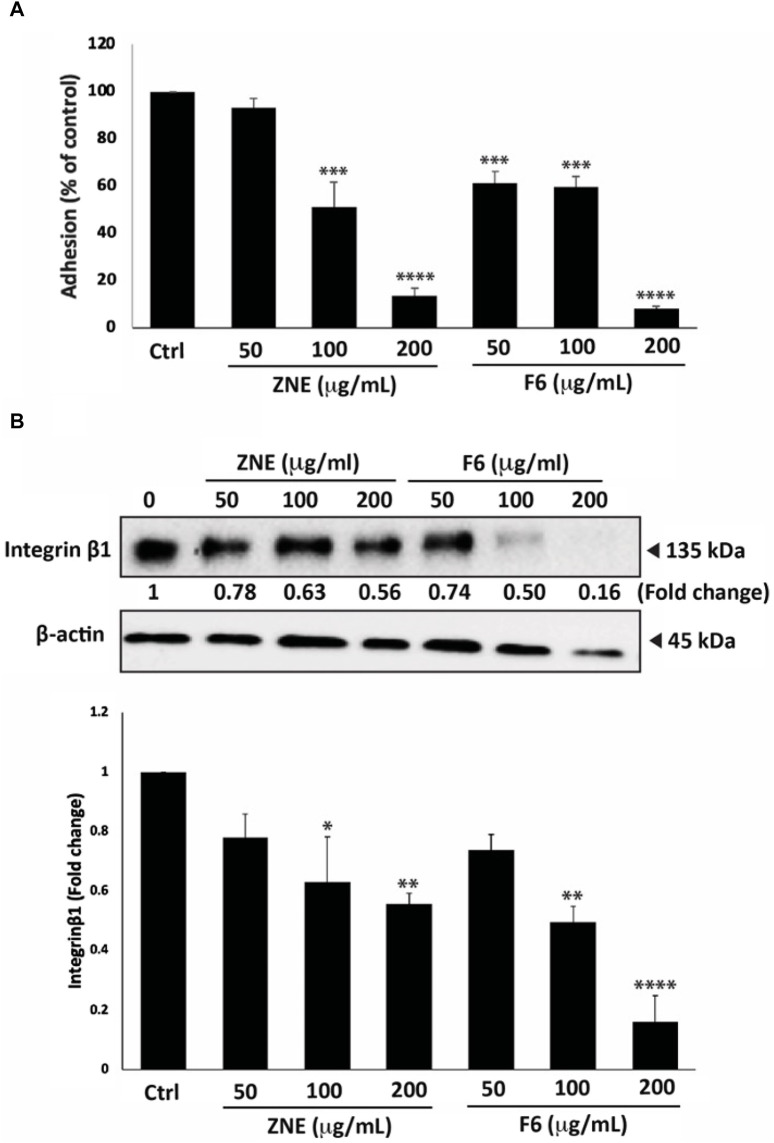
*Z. nummularia* extracts ZNE and F6 reduce the adhesion of MDA-MB-231 cells to collagen. **(A)** MDA-MB-231 cells were treated with the indicated concentrations of ZNE or F6 and then seeded onto collagen-coated cell culture wells and allowed to adhere for 1 h. Adhesion was determined using the MTT assay. Values are expressed as % of the vehicle-control and are represented as the mean ± SEM of three independent experiments. **(B)** MDA-MB-231 cells were treated with the indicated concentrations of ZNE or F6 for 24 h. Protein levels of integrin β1 were then determined using Western blotting. Values are expressed as fold change of the vehicle control and are represented as the mean ± SEM of three independent experiments. (*p < 0.05, **p < 0.01 ***p < 0.001, and ****p < 0.0001).

Cell adhesion to the extracellular matrix (ECM) is another crucial step for cell migration and cancer metastasis. ZNE and F6 considerably attenuated the ability of MDA-MB-231 cells to adhere to collagen, a key protein of the ECM (Figure 8A). The ZNE induced-reduction of cell adhesion to collagen was significant at 100 and 200 μg/mL, but not at 50 μg/mL. Whereas F6-induced reduction was significant at all three concentrations. Indeed, adhesion of cells treated with 200 μg/mL ZNE or F6 was decreased by 13.7 ± 3.0 and 7.8 ± 1.0-fold, respectively, compared to the vehicle-treated control cells.

Integrins play an integral role in facilitating the adhesion of invasive breast cancer cells to the ECM (Hamidi and Ivaska, 2018). Western blotting analysis showed that ZNE and F6 reduced the levels of integrin β1 in a concentration-dependent manner. The ZNE and F6 induced-reduction of integrin β1 protein levels was significant at 100 and 200 μg/mL, but not at 50 μg/mL. (Figure 8B). Taken together, these data suggest that the anti-adhesive effect of ZNE and F6 in MDA-MB-231 cells is mediated, at least partly, through collagen and integrin-β1.

### 3.9 ZNE and F6 induce cell-cell aggregation of MDA-MB-231 cells

**FIGURE 9:**
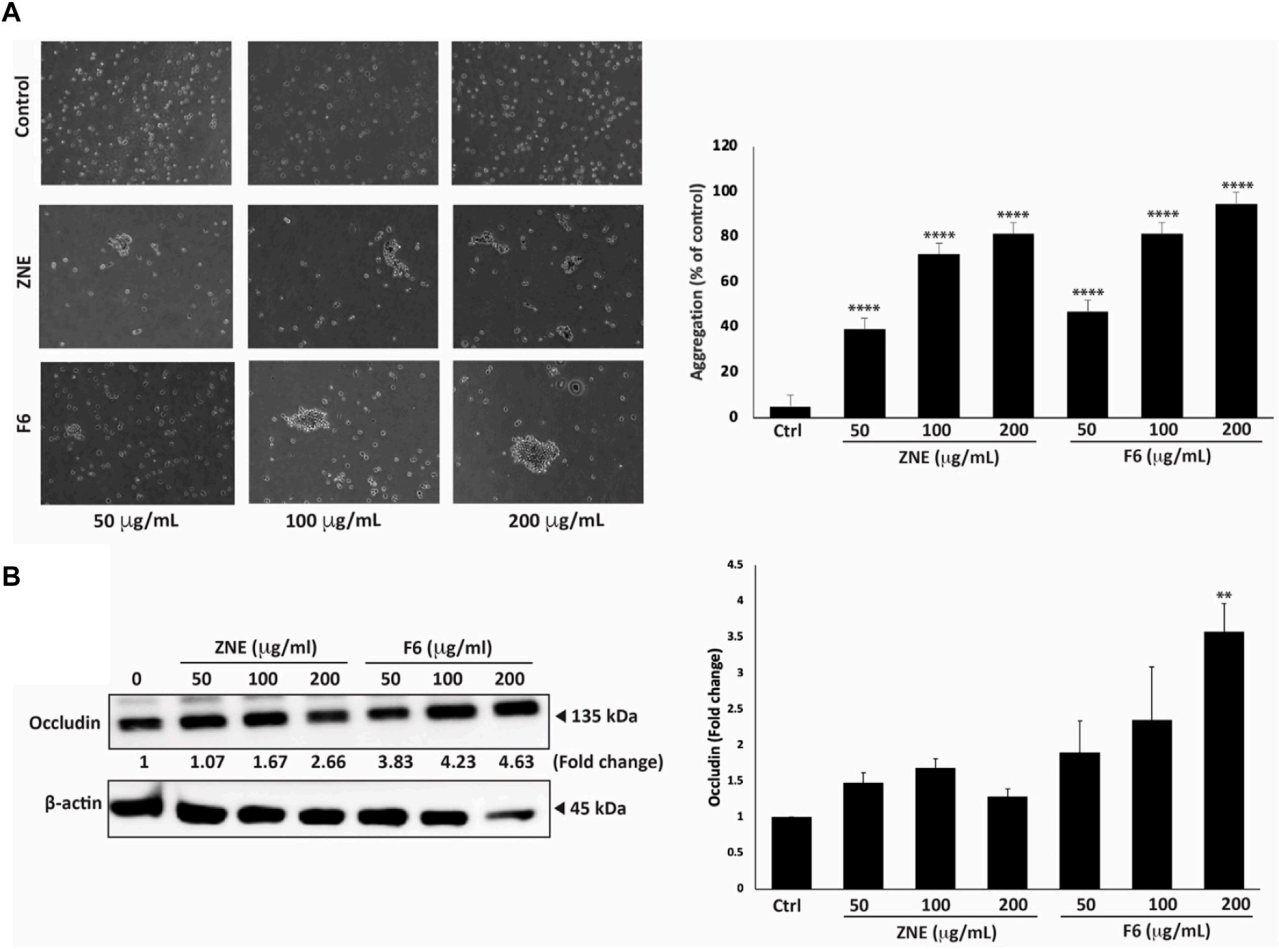
*Z. nummularia* extracts ZNE and F6 promote cell-cell homotypic adhesion of MDA-MB-231 cells. **(A)** MDA-MB-231 cells were treated with the indicated concentrations of ZNE or F6 and then subjected to an aggregation assay. The percentage of cell aggregation was calculated as: % aggregation = (1− Nt/Nc) × 100, where Nt and Nc represent the number of single cells in treated or control groups, respectively. Values are represented as the mean ± SEM of three independent experiments. **(B)** MDA-MB-231 cells were treated with the indicated concentrations of ZNE or F6 and incubated for 24 h. The protein levels of total Occludin were then determined using Western blotting. Values are expressed as fold change of the vehicle control and are represented as the mean ± SEM of three independent experiments. (**p < 0.01, and ****p < 0.0001).

Epithelial-mesenchymal transition (EMT) is a complex developmental process which is critical during cancer progression and metastasis (Kalluri and Weinberg, 2009). Using an assay of cell aggregation revealed that ZNE and F6 significantly induced MDA-MB-231 cell aggregation in a concentration-dependent manner at all the tested concentrations. In fact, the formation of MDA-MB-231 cell aggregates increased by 81.3% ± 3.7% and 94.6% ± 2.6% by a treatment of 200 μg/mL of ZNE or F6, respectively, suggesting that the extracts restore cell-cell adhesion (Figure 9A).

Expression of tight junction proteins, such as Occludins, is tightly controlled and its dysregulation impacts EMT, leading to cancer progression and metastasis (Osanai et al., 2006). Here, we showed that ZNE and F6 treatments increased total Occludin protein levels in a concentration-dependent manner. However, only 200 μg/mL of F6 significantly increased the levels of total Occludin proteins (Figure 9B).

## 4 Discussion

Plants and plant-derived metabolites still have major roles in the development of drugs for prevention or treatment of diseases. Plant secondary metabolites have a variety of pharmacological properties, and many have been used as leads for the synthesis of conventional drugs (Seca and Pinto, 2018). Many of the current cancer chemotherapeutic agents are in origin plant-derived drugs. For instance, vincristine, one of the first drugs to be approved by the Food and Drug Administration (FDA) to treat several cancers including lymphoblastic leukemia, neuroblastoma, and nephroblastoma, was isolated from leaves of *Catharanthus roseus* (Škubník et al., 2021). Paclitaxel, isolated from the bark of *Taxus brevifolia*, is one of the most effective drugs used in the treatment of breast, ovarian, and lung cancers (Škubník et al., 2021). Currently, there is a renewed interest in screening plants for therapeutic targets. This is partly driven by the fact that several herbal remedies have been shown to have efficacy and low toxicity therapy of diseases (Konkimalla and Efferth, 2008; Lee et al., 2021). Moreover, plant secondary metabolites are being employed as scaffolds for developing more potent chemotherapeutic agents (Shaito et al., 2020b; Nisar et al., 2022). Many studies have reported the promising pharmacological properties of plant-based metabolites in *in vitro* in cell lines and *in vivo* in animal models. Plant based remedies have antioxidant, apoptosis-promoting effects, and can attenuate cell proliferation, metastasis (cell aggregation, migration, adhesion), and angiogenesis (Park et al., 2021; Anwar et al., 2022). Of particular interest to this study, there are very few studies (Dey Ray and Dewanjee, 2015; Beg et al., 2016; Mesmar et al., 2021) on the anti-cancerous properties of the shrub *Z*. *nummularia*, despite that this plant has been shown to have numerous therapeutic uses in traditional medicinal cultures in the countries where it is indigenous (Chopra and Nayar, 1956; Upadhyay et al., 2011; Abbasi et al., 2013; Dey Ray et al., 2015; Hussain et al., 2017; Golla, 2018; Muhammad et al., 2020; Mesmar et al., 2022a). Furthermore, previous, analyses of *Z. nummularia* phytochemical composition have revealed around 431 chemical constituents which belong to all phytochemical classes (Kumar et al., 2002; Bodroth and Das, 2012; Sonia and Singh, 2019; Zandifar et al., 2020; Mesmar et al., 2022a), suggesting that it is pertinent to investigate the anticancerous properties of this plant. We commenced to evaluate the anticancerous properties of *Z. nummularia in vitro* in TNBC cells, which do not respond to hormonal therapies and often develop resistance to chemotherapy.

Here we show that an ethanolic extract of *Z. nummularia* (ZNE) has promising antitumor effects against MDA-MB-231 human TNBC cells. ZNE effectively inhibited the proliferation, migration, and invasion, and enhanced the aggregation of MDA-MB-231 cells, validating its potential as a promising source of phytochemicals with anticancer properties. Notably, we chromatographically fractionated ZNE and one of its fractions, F6, showed enhanced anticancerous effects over the crude ZNE, or any of its other fractions (For instance, the IC_50_ at 48 h for F6 was 49.1 μg/mL, in contrast to 60.8 μg/mL for ZNE). Consequently, we investigated the chemical composition of ZNE and F6 and were able to identify chemicals that may be responsible for ZNE anticancerous effects. Qualitative phytochemical analysis of ZNE and F6 showed that ZNE and F6 had high TFC and TPC values. This indicated that ZNE and F6 are enriched in polyphenolic metabolites and flavonoids, in agreement with previous reports (Kumar et al., 2002; Bodroth and Das, 2012; Sonia and Singh, 2019; Zandifar et al., 2020). This finding is relevant, especially that therapeutic and antioxidant properties of plant extracts are mostly attributed to flavonoids and polyphenols (Bhosale et al., 2020; Seglab et al., 2021; Slika et al., 2022). Natural polyphenols are plant secondary metabolites with two or more phenol rings. Polyphenols are reported to have antioxidant, antidiabetic, cardioprotective, neuroprotective effects. They are also anticancer agents that can inhibit cell cycle progression, induce apoptosis, inhibit metastasis (Bhosale et al., 2020). Flavonoids are natural polyphenols with documented anticancer and antioxidant properties (Seglab et al., 2021; Slika et al., 2022). As expected, the TFC and TPC values of ZNE were higher than those of F6, in agreement with the results of the HPLC-PDA-MS/MS, which revealed that F6 is partially purified and had less polyphenolic and flavonoid metabolites. Of note, the TFC values are lower than the TPC values for both ZNE and F6, which is reasonable since flavonoids are a subclass of natural polyphenols.

Analysis of chemical composition of ZNE and F6 by HPLC-PDA-MS/MS revealed a difference in the chemical profile between F6 and ZNE; F6 had far less phytochemicals (31 metabolites) than ZNE (76 metabolites), indicating that F6 has a better specific activity and is more pure than ZNE (F6 has less IC_50_ and less phytochemicals). Caffeic acid and its derivatives are widely present in fruits and grains and have been associated with a variety of pharmacological activities including antioxidant, anti-inflammatory, neuroprotective (Spagnol et al., 2019; Alam et al., 2022; Pavlíková, 2022), and, relatedly, anticancerous activities against several types of cancers such as human renal carcinoma (Jung et al., 2007), colon cancer (Kang et al., 2011), and breast cancer (Rosendahl et al., 2014; Serafim et al., 2015; Kabała-Dzik et al., 2017). However, it is worth mentioning that in a plant extract, oftentimes, the synergy between phytochemicals of the extract is the one which confers the pharmacological activities such as improving the efficacy or overcoming resistance to drugs.

HPLC-PDA-MS/MS also identified the chemical constituents of ZNE and F6, and several of these phytochemicals have been previously reported to have anticancer properties. As examples, quercetin dirhamnoside has been reported to have cytotoxic activity on HeLa cervical cancer cells (Herni Kusriani et al., 2021). Coumaric acid exhibited antitumor effects on melanoma (A375 and B16 cells) (Hu et al., 2020) and colorectal cancer cells (Tehami et al., 2023). Kaempferol rhamnoside has been shown to inhibit MCF-7 breast cancer cell proliferation through the activation of the caspase cascade pathway (Diantini et al., 2012). This warrants further investigation into the activity of these metabolites and their molecular mechanisms of action.

ZNE and F6 significantly attenuated cell proliferation of MDA-MB-231. This is the first report to show anticancerous effects of *Z. nummularia* in a TNBC cell line (Dey Ray and Dewanjee, 2015; Beg et al., 2016; Mesmar et al., 2021). This is the second study to investigate the anticancerous properties of leaves of *Z. nummularia*. Previous reports on the antiproliferative effects of *Z. nummularia* used root bark extracts (Dey Ray and Dewanjee, 2015), or a methanolic extract of the fruit (Beg et al., 2016), and only one study tested leaves extracts in human pancreatic cancer cells (Mesmar et al., 2021). In confirmation, the levels of the proliferation marker Ki67, which is highly expressed in TNBC and linked to its aggressive characteristics (Yang et al., 2018), were significantly decreased by ZNE and F6 treatment. The decrease in Ki67 levels further reaffirms the potential *Z. nummularia* as a promising avenue for the development of therapeutic agents targeting TNBC. ZNE and F6 exerted their antiproliferative effects at least partly by arresting the cell cycle at the at the G_1_ phase. Other *Ziziphus* species, mainly *Zizyphus jujuba* and *Ziziphus spina-christi,* have been reported to induce G_0_/G_1_ cell cycle arrest in HepG2 and MCF-7 cells, respectively (Huang et al., 2007b; Farmani et al., 2016). Mechanistically, *Z. nummularia* extracts, ZNE and F6, activated p38, which is a stress-induced kinase involved in maintaining cellular homeostasis. Importantly, p38 acts as a tumor suppressor by inducing cell cycle arrest and apoptosis (Cuenda and Rousseau, 2007; Loesch and Chen, 2008; Taylor et al., 2013; Athamneh et al., 2020). Our results are in line with the reported events of p38 activation, where ZNE and F6 decreases the viability of MDA-MB-231 cells, induced a cell cycle arrest, and activated p38. ZNE and F6 also increased the levels of the cell cycle inhibitor p21 and p27. These are downstream targets of p38 and are usually dysregulated in many cancers and as such can be used as targets for the design of anticancer therapeutics. This result further attests to the activation of p38 signaling by ZNE and F6. Rb is another downstream effector of p38 that is activated by ZNE and F6. Rb, a tumor suppressor that controls the G_1_/S phase of the cell cycle and plays a key role in the proliferation of normal cells. Studies have shown that the inactivation of Rb can cause cancer formation. Phosphorylation of Rb by p38 delays cell cycle progression (Gubern et al., 2016). Contextually, ZNE and F6 significantly induced the phosphorylation of Rb, further implicating the p38 MAPK pathway in the proliferation of MDA-MB-231 cells. Future investigations, using specific p38 inhibitors or genetic knock out of p38 in MDA-MB-231 cells are needed to show if p38 activation is necessary for ZNE and F6 effects in MDA-MB-231 cells.

In addition to blocking proliferation, the p38 MAPK pathway promotes cell death through apoptosis. Here we showed that *Z. nummularia* induced intrinsic apoptosis as observed by the cleavage of Caspase 3, the reduction of the anti-apoptotic BCL-2 protein, and induction of the pro-apoptotic BAX protein. This is similar to results reporting the effect of *Z. nummularia* on human pancreatic cancer cells (Mesmar et al., 2021), and similar to results obtained with other Ziziphus species like *Ziziphus jujube* (Abedini et al., 2016; Pillai, 2020) and *Z. spina-christi* (Ghaffari et al., 2021).

TNBC cells often manage to evade apoptosis and develop resistance to chemotherapy (Chakrabarty et al., 2021). In such instances where tumor cells evade apoptosis, autophagy emerges as an alternative pathway for cell death (Towers et al., 2020). Autophagy is a crucial process to regulate cell growth and homeostasis, but its over-activation leads to cell death. In the case of cancer, autophagy has been reported to play dual roles, depending on the stage of the tumor (Li et al., 2020). For instance, autophagy prevents tumor initiation by acting as a survival pathway and ensuring quality control. Whereas it contributes to the growth invasiveness of cancer cells that have progressed into late stages. In this study, we showed that ZNE or F6 induced autophagy by the accumulation of the main autophagy protein marker LC3B. This is in agreement with results reported for other plant extracts which induced both apoptosis and autophagy in various cancer cell lines (Lin et al., 2007; Athamneh et al., 2017; Gao et al., 2017; Athamneh et al., 2020).

ROS play crucial roles as signaling molecules, and have been reported to have pro-tumorigenic as well as anti-tumorigenic effects (Huang R. et al., 2021). At low concentration, ROS carry out vital signaling functions and are needed to maintain cellular homeostasis. At high concentrations, they can damage cellular molecules including lipids, proteins, and genomic DNA, and thus can lead to tumor initiation. In fact, ROS have been implicated in tumor invasion and metastasis as well. On the contrary, high concentrations of ROS can inhibit tumor growth by blocking cancer cell proliferation and inducing cell death. In the same context, chemotherapy and radiotherapy can eliminate cancer cells mainly by elevating the levels of intracellular ROS (Khan et al., 2021). In our study, antioxidant free-radical-scavenging capacity of ZNE in the DPPH assay was moderate (IC_50_ = 373.13 μg/mL) while that of F6 was strong (32.88 μg/mL); F6 free-radical-scavenging IC_50_ is on par with that of ascorbic acid, which suggests that the purification step has enriched F6 with antioxidant molecules. However, both ZNE and F6 significantly induced ROS formation in MDA-MB-231 cells, indicating that ZNE and F6 did not use their free radical scavenging potential inside the cell, but rather acted through mechanisms that increased ROS generation. In this study, suppressing ROS levels by using a strong antioxidant such as NAC attenuated ZNE- and F6-induced reduction of viability of MDA-MB-231. Collectively, this suggests a scenario where ZNE and F6 caused an increase in ROS levels triggered anti-proliferative signaling pathways in MDA-MB-231 cells. It is plausible that the ROS-p38 axis is involved in this signaling. Activation of p38 signaling axis has been found to play a pivotal role in tumor initiation, proliferation, and suppression by acting as a sensor for oxidative stress (Kennedy et al., 2007). Similarly, previous studies have shown that ROS inhibition attenuated the anti-proliferative effect of *Z. nummularia* in human pancreatic cancer cells (Mesmar et al., 2021). Similar findings were reported for *Z*. *jujuba*, which increased ROS levels and led to a decrease in cell viability of HepG2 human liver cancer cells (Huang et al., 2007a). These results also align with studies which demonstrated that other plant extracts reduced the viability of cancer cells by elevating oxidative stress through inhibition of cellular antioxidant systems (Yedjou and Tchounwou, 2012; Iwasaki et al., 2016; Li et al., 2017; Alsamri et al., 2019). Future experiments, using both p38 inhibitors and ROS scavengers, should reveal the mode of interaction between ROS and p38; whether p38 is acting upstream or downstream of ROS or if there exists a bidirectional crosstalk.

Cancer metastasis is the major cause of treatment failure and cancer-related deaths. Patients with metastatic TNBC have a very poor prognosis (Bianchini et al., 2016). Cell migration is required for successful metastasis. Physiologically, cell migration is needed for may processes such as tissue formation, wound healing, and immune responses. Deregulation of cell migration is an early step of cancer metastasis. Migration of cells includes the down expression of proteins of junctional and adhesion complexes and degradation of surrounding ECM by matrix metalloproteinases (MMPs) (Singh and Settleman, 2010; Trepat et al., 2012). We assessed the effect of ZNE and F6 on migration and invasion abilities of MDA-MB-231 cells using a wound/scratch healing assay and Boyden chamber assays. The findings showed that both ZNE and F6 inhibited MDA-MB-231 cell migration and invasion, with F6 showing more enhanced effects than the crude extract. Once cancer cells have migrated and adhered to secondary tissues, invasion processes are initiated. Degradation of the ECM, by proteinases particularly MMPs, is not only required for cell migration and invasion at the initial stages of metastasis where the primary tumor cells need to extravasate, but also at secondary tumor sites where the tumor cells need to intravasate to secondary tumor sites (Saliba et al., 2023; Shaito et al., 2023). In particular, the protease MMP-9 is overexpressed in breast cancer and its expression is associated with a higher incidence of metastasis, and therefore it is used as a prognosis marker (Joseph et al., 2020). Here we found that ZNE and F6 significantly reduced MMP-9 activity in MDA-MB-231. It is plausible that inhibition of MMP-9 activity is responsible for the compromised migratory and invasive capabilities induced by ZNE and F6. Other plant extracts have been reported to attenuate the migration and invasion of cancer cells by acting through MMP-2 and MMP-9 reduction (AlKahlout et al., 2022).

Deregulation of EMT pathways is another major hallmark of tumor to metastasis being required for primary tumor cells to migrate and invade secondary sites. EMT deregulation includes dysregulation of proteins that are part of the cell-cell interactions as well as cell-ECM complexes (Savagner et al., 2005; Singh and Settleman, 2010). For example, the reduction of epithelial markers such as E-cadherin, and tight junction proteins including Occludins, is associated with advanced metastatic stages in patients with breast cancer (Martin et al., 2010; Kyuno et al., 2021). In our study, we showed that ZNE and F6 strongly induced cell-cell homotypic adhesion of MDA-MB-231 cells. This was associated with a rise in levels of total Occludins, indicating that Z*. nummularia* extracts may disrupt tumor migration by enhancing cell-cell adhesion.

At distant secondary sites of metastasis, cancer cells have an enhanced ability to adhere to the ECM, which facilitates their intravasation process (Huang J. et al., 2021). Therefore, reducing cell-ECM adhesion would ultimately reduce successful tumor metastases. Here, we showed that ZNE and F6 attenuated cell adhesion to a key component of ECM, collagen I. This action maybe mediated by the ZNE- and F6-mediated reduction of integrin-β1 protein levels in TNBC cells. This is in line with other studies which have shown that plants extracts have reduced cell-ECM adhesion and thus inhibited the metastatic potential of TNBC cells (El Hasasna et al., 2016; AlKahlout et al., 2022; Mesmar et al., 2022b). These results invite future investigation of the ability of *Z. nummularia* to impact other EMT proteins such as E-cadherin, N-cadherin, vimentin, Slug, Snail, and others (Singh and Settleman, 2010; Thiery and Lim, 2013; El-Hajjar et al., 2019; AlKahlout et al., 2022; Mesmar et al., 2022b).

Despite the significant findings, this study has several limitations. Further analysis and purification of F6 is needed to isolate the bioactive metabolites responsible for the observed anticancerous properties of ZNE. In addition, the study did not confirm the observed *in vitro* effects of ZNE or F6 *in vivo* in an animal model of TNBC. This would provide valuable insights into the efficacy of ZNE and its metabolites as anticancer agents. In fact, often time plant extracts or phytochemicals do not show *in vivo* efficacy despite their potent *in vitro* effects. Despite the promise of phytochemicals in TNBC therapy, they still have several limitations that hinder their clinical application for TNBC therapy (Alaouna et al., 2023). Future investigations should be directed to uncover the anti-TNBC potential of ZNE and F6 in an *in vivo* animal model. Lastly, many environmental factors affect the phytochemical composition, and therefore biological activity, of medicinal plants including soil type, seasonal variation, salinity, light radiation, grazing stress, altitude, humidity and other agro-climatic factors. These variations dictate characterization of the best time/season/location to harvest individual plant species to guarantee an optimal phytochemical composition rich in the sought after medicinal properties (Iqbal and Bhanger, 2006; Nchabeleng et al., 2012; Kumar et al., 2017a; Kumar et al., 2017b). Variation in temperature significantly affects antioxidant content especially in extreme stress conditions such as cold or hot weather (Iqbal and Bhanger, 2006). Cooler climates lead to higher production of unsaturated fatty acids, phenolics, and other antioxidants as a self-defense mechanism against environmental stress (Sanders, 1982; Kumar et al., 2017a; Kumar et al., 2017b). Altitude was also shown to alter the level of flavonoids, phenolic acid, steroids, terpenes, and allantoin in *Epilobium hirsutum* (Mohammadi Bazargani et al., 2020). In arid environments, higher temperatures produced more phenolic compounds and antioxidants in *Calligonum polygonoides L.* (Berwal et al., 2021). The effect of geographical and environmental factors on phytochemical composition of plants is so profound that a machine learning model was designed to predict, with decent accuracy, phytochemical abundance in different eco-climatic zones and elevations (Tiwari et al., 2023). Defossez *et al.* screened the metabolome and therefore characterized the phytochemical diversity of 416 vascular plant species growing at different elevations. The authors demonstrated that combining phylogenetic information, topographic, edaphic, and climatic variables, can predict phytochemical diversity of plant communities (Defossez et al., 2021). In our study, we used *Z. nummularia*, from Qatar, a country characterized by a very hot arid environment. It is possible that the temperature and humidity stresses imposed by the Qatari environment have resulted in a unique phytochemical composition in the Qatari *Z. nummularia*. This notion need to be tested using *Z. nummularia* leaves from areas with similar arid environments such as the nearby Saudi Arabia and *Z. nummularia* leaves from areas with less climate stresses such as Iran or Lebanon.

In conclusion, our study indicates that the ethanolic extract of leaves *Ziziphus nummularia* is rich in important plant phytochemicals, many of which have reported pharmacological activities. We also demonstrated that the extract and its fraction F6 possess strong anti-cancer and anti-metastatic properties that may be able to attenuate the malignant phenotype of TNBC. The extract and F6 impacted hallmarks of the carcinogenesis process, including cell proliferation, cell cycle regulation and cellular adhesion, migration, and invasion. Fraction F6 of the extract was more pure and, in most assays, more effective than the crude extract. This warrants further analysis and purification of F6, to further purify and isolate its bioactive metabolites, responsible for the observed anticancerous properties. Such studies may place *Ziziphus nummularia* as a new source of novel leads for drug discovery of promising therapeutics for TNBC and other cancers.

## Data Availability

The raw data supporting the conclusion of this article will be made available by the authors, without undue reservation.
