# Supplementary material for: Fractionation and phytochemical composition of an ethanolic extract of Ziziphus nummularia leaves: antioxidant and anticancerous properties in human triple negative breast cancer cells
Source: Front Pharmacol. 2024 Feb 9;15:1331843. doi: 10.3389/fphar.2024.1331843 (PMC10885810; doi:10.3389/fphar.2024.1331843)
Supplement: Supplementary file 1 [file DataSheet1.pdf]

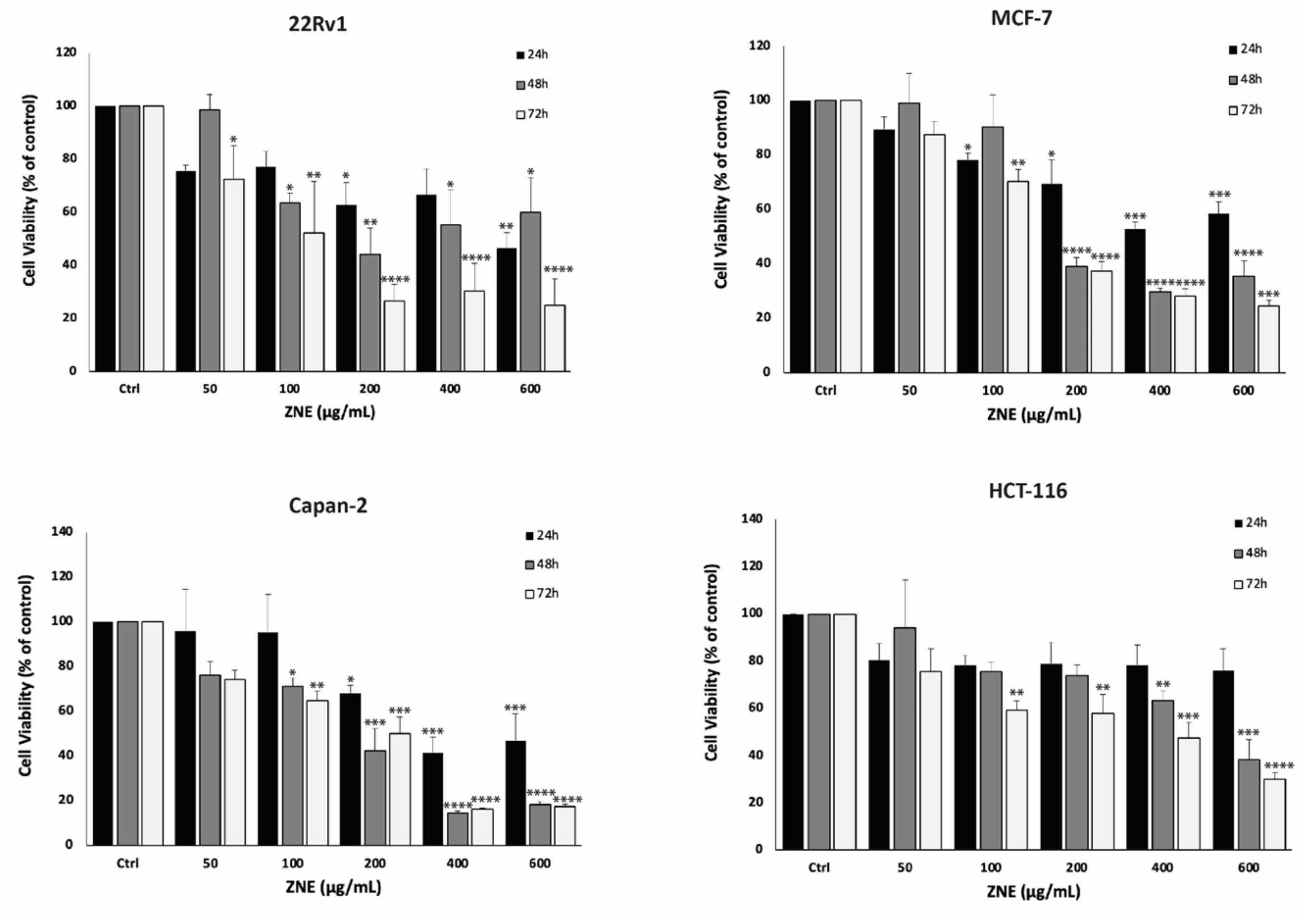

	IC50 at 48h (µg/ml)	IC50 at 72h (µg/ml)
22RV1	126.027	96.049
Capan-2	157.7	150.2
HCT-116	485.92	266.99
MCF-7	163.063	150.49

Figure S1. *Z. nummularia* leaves ethanolic crude extracts inhibit proliferation of 22RV1 prostate cancer, Capan-2 pancreatic cancer, HCT-116 colon cancer, and MCF-7 breast cancer cells. (**A**) Cells were obtained from ATCC and grown in the appropriate cell culture media at 37OC in a humidified cell culture incubator. Cells were treated the indicated concentrations of ZNE for 24, 48 and 72 h. Cell viability was determined using an MTT assay. Values are expressed as % viability compared to vehicle-treated control and are represented as the mean ± SEM of three independent experiments. *$p < 0.05$, **$p < 0.01$, ***$p < 0.001$, and ****$p < 0.0001$. The lower Table shows the IC50 of inhibition of proliferation of the different cell lines by ZNE at 48 h and 72 h.
